# The Antibacterial Effects of Resin-Based Dental Sealants: A Systematic Review of In Vitro Studies

**DOI:** 10.3390/ma14020413

**Published:** 2021-01-15

**Authors:** Saad Saeed AlShahrani, Mana’a Saleh AlAbbas, Isadora Martini Garcia, Maha Ibrahim AlGhannam, Muath Abdulrahman AlRuwaili, Fabrício Mezzomo Collares, Maria Salem Ibrahim

**Affiliations:** 1 College of Dentistry, Imam Abdulrahman Bin Faisal University, Dammam 31441, Saudi Arabia; 2150008724@iau.edu.sa (S.S.A.); 2160006146@iau.edu.sa (M.S.A.); 2170001326@iau.edu.sa (M.I.A.); 2160004214@iau.edu.sa (M.A.A.); 2 Dental Materials Laboratory, Department of Conservative Dentistry, School of Dentistry, Federal University of Rio Grande do Sul, Porto Alegre 90035-003, Brazil; isadora.garcia@ufrgs.br (I.M.G.); fabricio.collares@ufrgs.br (F.M.C.); 3 Department of Preventive Dental Sciences, College of Dentistry, Imam Abdulrahman Bin Faisal University, Dammam 34212, Saudi Arabia

**Keywords:** sealant, antibacterial, caries prevention

## Abstract

This review aimed to assess the antimicrobial effects of different antibacterial agents/compounds incorporated in resin-based dental sealants. Four databases (PubMed, MEDLINE, Web of Science and Scopus) were searched. From the 8052 records retrieved, 275 records were considered eligible for full-text screening. Nineteen studies met the inclusion criteria. Data extraction and quality assessment was performed by two independent reviewers. Six of the nineteen included studies were judged to have low risk of bias, and the rest had medium risk of bias. Compounds and particles such as zinc, tin, Selenium, chitosan, chlorhexidine, fluoride and methyl methacrylate were found to be effective in reducing the colony-forming unit counts, producing inhibition zones, reducing the optical density, reducing the metabolic activities, reducing the lactic acid and polysaccharide production and neutralizing the pH when they are added to the resin-based dental sealants. In addition, some studies showed that the antibacterial effect was not significantly different after 2 weeks, 2 months and 6 months aging in distilled water or phosphate-buffered saline. In conclusion, studies have confirmed the effectiveness of adding antibacterial agents/compounds to dental sealants. However, we should consider that these results are based on laboratory studies with a high degree of heterogeneity.

## 1. Introduction

Dental caries is a highly prevalent chronic disease, affecting more than 60% of school children [1,2]. Caries is a biofilm-sugar-dependent disease. The bacteria in the biofilm over the teeth metabolize fermentable carbohydrates and produce acids [3]. The acids demineralize the hydroxyapatite (HAp) of dental tissues (enamel and dentin), leading to an irreversible process over time [3].

The loss of minerals is considered as an unbalance in the natural demineralization-remineralization process of teeth, and it causes caries lesions to have several stages. In the early stages, carious lesions have subclinical characteristics [4]. However, if the biofilm persists over the teeth due to poor oral hygiene and frequent consumption of fermentable carbohydrates such as sucrose, there will be a periodic reduction in the pH of the oral environment [4]. In such scenarios, dental caries becomes clinically visible in the form of white-spot lesions [4]. Initial non-cavitated white lesions may become cavitated if the process of demineralization continues without the process of remineralization. Although these carious lesions usually get restored, recurrent caries could appear around the restorations [5]. Each time the restoration is replaced, dental tissue is eventually removed. This process, identified as “tooth death spiral”, can lead to total tooth loss [6]. Therefore, prevention and intervention at the beginning of the disease are essential to prevent teeth loss.

Besides being a sugar-dependent biofilm, dental caries is influenced by the individual’s behavior, education and social class. These factors can modify the severity of the disease [5]. Because of the multifactorial nature of caries, it is important to assess the susceptibility of each individual patient to dental caries and decide which preventive measures could be used in each case [7,8]. Acidic gels with high fluoride concentrations are often used topically to treat initial lesions since fluoride—in biofilm and oral fluids—helps to inhibit the demineralization process and induce the remineralization of teeth [9]. Moreover, diet and hygiene instructions must always be present as part of caries prevention measures. In addition to these measures, the application of light-polymerizable resin-based sealants to mechanically seal dental surfaces is a procedure with a reasonable success rate in preventing occlusal caries [7].

Dental sealants are used mainly for sealing occlusal surfaces, which are highly susceptible to biofilm accumulation [7]. Sealants are commonly used right after the full eruption of the first permanent molars around six years of age. Sealants have been reported to reduce the incidence of caries by around 80% among children at high caries risk [7]. However, around 20% of children at a high-caries risk still develop new lesions even with dental sealants [7]. Therefore, improvements in the composition of sealants have been suggested to increase their efficacy.

Traditional sealants in the market are based on methacrylate resins with no or little bioactivity such as antibacterial or remineralizing properties. They are usually composed only of conventional monomers, such as bisphenol glycidyl methacrylate (BisGMA) and triethylene glycol dimethacrylate (TEGDMA), and inorganic particles such as fluoro-aluminosilicate glass powder [10,11,12,13]. Due to the well-known action of fluoride against dental caries [14], some manufacturers have added this element in the composition of sealants [7]. However, until now, the addition of fluoride in sealants does not seem to have a remarkable effect on preventing caries progression or reducing the incidence of new carious lesions [7]. The reason for that is probably because fluoride will be released over time from the resin matrix due to its lack of binding to the polymer [15]. Other compounds such as sodium monofluorophosphate, bioglasses and nanoamorphous calcium phosphate were also investigated to prevent caries at the sealant-tooth interface [16,17,18,19].

Recently, research has focused on the improvement of antibacterial activity that sealants could present [20,21,22,23]. These agents would benefit from providing an improved therapeutic action to the material by reducing the chance of bacteria colonization and biofilm formation at the tooth-sealant interface. This approach may reduce the incidence of new lesions in the teeth of children at high risk of caries. There is a wide range of tests applied to evaluate the antibacterial activity of these materials. However, no studies summarized the information available to date in the literature on sealants with antimicrobial agents. This review aimed to identify the antibacterial agents incorporated into resin-based dental sealants and analyze which methods were applied to evaluate the performance of these materials. In addition, the review aimed to evaluate the quality of evidence.

## 2. Materials and Methods

### 2.1. Research Question

The main review question was what are the antibacterial effects of resin-based dental sealants that incorporate antibacterial agents in their structure? The review followed the Preferred Reporting Items for Systematic Reviews and Meta-Analyses (PRISMA) guidelines for systematic reviews and meta-analysis [24]. The review protocol was pre-determined but not published.

### 2.2. Search Strategies

The search strategies of the four electronic databases are described in Table 1. They were developed and applied by three authors (M.S.A, M.I.A. and M.S.I.). The last search was run on 1 June 2020. No date or language restriction was applied at this stage. The resulting citations from all databases were imported to Covidence online platform for screening.

### 2.3. Inclusion and Exclusion Criteria

Studies included in this review were in vitro, laboratory studies that assessed the antimicrobial activities of resin-based dental sealants. Studies that are not laboratory studies, studies that did not have resin-based sealants, studies that only tested resin-modified glass ionomer and studies that did not assess any antibacterial activity were excluded. The compositions on commercial resin-based sealants were searched through the safety data sheet of the material itself or the literature to confirm that the sealant is a resin-based material.

### 2.4. Studies Screening and Selection

Selection of the studies was performed using a pair of reviewers (M.S.A., M.I.A. and M.A.A.). Reviewers were not blinded to the identity of the authors or journal. The selection process consisted of title and abstract screening then full text screening. If any of the exclusion criteria were found, the record was excluded. Disagreements among reviewers were solved by a senior reviewer (M.S.I.).

### 2.5. Data Extraction

Two reviewers (S.S.A and M.S.I.) extracted data regarding various variables from the included studies using customized data collection forms. The extracted data included qualitative and quantitative data. The following data were extracted: the details of the sample including sample size, type, measurements and curing time, assessment methods used to assess the antibacterial effect, other properties assessed beside antibacterial properties, the antibacterial agents, bacteria or type of inoculum used and the control and tested (interventional) groups details. In addition, data about the outcome measures, in regard to units and effects, were extracted.

### 2.6. Quality Assessment

The risk of bias of the included studies was assessed by three independent reviewers (S.S.A, M.S.A and M.S.I.). The assessment tool was adapted from previously published scoping and systematic reviews [25,26]. Studies with one to three “Yes” only were considered to have a low risk of bias. Studies scoring four to six “Yes” or seven to nine “Yes” were considered to have a medium risk of bias or a high risk of bias, respectively.

### 2.7. Assessment of Heterogeneity

Two reviewers (S.S.A. and M.S.I.) extracted data about the interventional, methodological and statistical heterogeneities of the included studies. Interventional heterogeneity was assessed by checking the differences in compositions of the tested groups and control groups among the included studies, such as the percentage and type of the fillers and incorporation of other agents such remineralizing agents. Methodological heterogeneity was assessed by extracting data in regard to the sample types, curing time, bacteria type or inoculum source, the methods of outcome assessment and the study’s overall risk of bias.

### 2.8. Data Synthesis

A qualitative summary of the assessment methods, interventions, outcomes and any additional relevant information was planned to be reported. We also planned to perform a quantitative meta-analysis using a fixed-effect model or a random-effect model if an I_2_ statistics at or below 50% was found with no significant methodological heterogenicity or an I_2_ statistics was found to be above 50% with no significant methodological heterogenicities, respectively. On the other hand, if a significant statistical heterogeneity or a methodological heterogenicity was found, a meta-analysis was not planned to be conducted.

## 3. Results

### 3.1. Studies Selection

**Figure 1:**
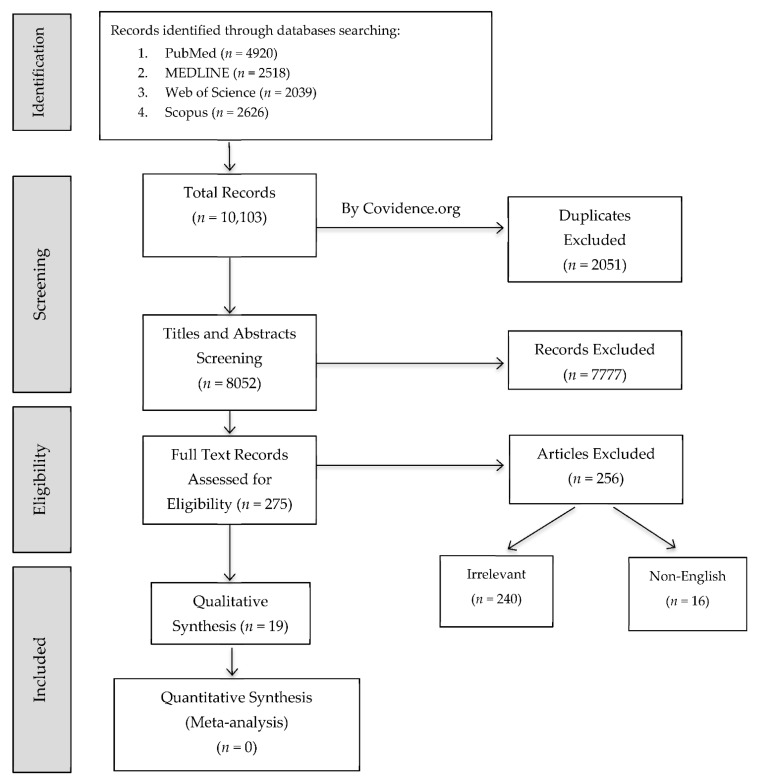
Flow diagram of study screening and selection.

A total number of 10,103 potentially relevant records were retrieved from the four databases. Duplicates were initially removed by Covidence.org and 8052 records included for abstract and title screening. Two hundred and seventy-five records were eligible for full-text screening. Nineteen records met the inclusion criteria and were included in the review. The screening process is reported in Figure 1.

### 3.2. Risk of Bias Appraisal

**Figure 2:**
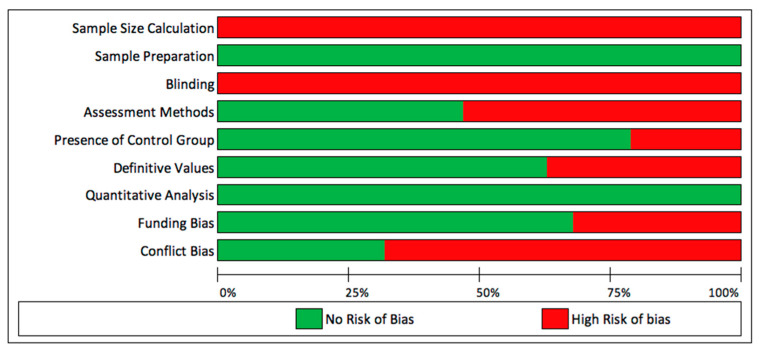
Overall risk of bias for each parameter.

Out of the 19 included studies, six were judged to have low risk of bias, and the rest were of medium risk of bias (Table 2). Sample size calculation and blinding were not reported in all of the included studies, which led to a positive risk of bias in these two parameters (Figure 2). Studies always reported the sample preparation or measurements and there was always at least one method of quantitative assessment (Figure 2).

### 3.3. Studies Characteristics

A summary about the characteristics of the 19 included studies is reported in Table 3.

#### 3.3.1. Samples

Out of the 19 included studies, only one study [29] used human teeth samples and one study used bovine teeth samples [37], while most of the studies used discs samples of the same material. There was a large variation between the studies in regard to the number of samples used to assess the antibacterial effect. This variation was also found between different tests in the same study. The light-curing/polymerization time was reported in most of the studies and showed variation. However, five studies [10,13,32,39,40] did not report any specific time.

#### 3.3.2. Antibacterial Agents/Compounds

Studies investigated various antibacterial agents/compounds including zinc methacrylate (ZnM), tin methacrylate (SnM), zinc oxide (ZnO), organo-selenium, chitosan, chlorhexidine (CHX), calcium fluoride (CaF_2_), 2-methacryloxylethyl dodecyl methyl ammonium bromide (MAE-DB), 2-methacryloyloxyethyl trimethylammonium chloride (METAC), methacryloxylethyl cetyl dimethyl ammonium chloride (DMAE-CB), dimethylaminohexadecyl methacrylate (DMAHDM) and 1,3,5-triacryloyl hexahydro-1,3,5-triazine (TAT) (Table 3). Fluoride was the most commonly incorporated agent in different studies [28,36,37,38,39,40].

#### 3.3.3. Bacteria and Inoculum

Most studies (17 studies) tested the antibacterial activities of the sealants against *S. mutans*. One study [21] tested the effect against multispecies biofilms cultured from pooled saliva of high-caries risk and low-caries risk pediatric dental patients, and another study tested the effect against *Enterococcus faecalis* [29]. Other bacteria such as *L. acidophilus* [28,32,37,38], *S. oralis* [10], *C. albicans* [10], *S. salivarius* [13] and *S. sobrinus* [40] were also used to test the antibacterial effects.

#### 3.3.4. Antibacterial Activity Assessment Methods

Studies investigated the antibacterial effects of the studied sealants using various assessment methodologies and tests (Table 3). The most common assessment method was a colony forming unit (CFU) counting test [10,11,12,13,21,27,28,30,34,35], followed by an inhibition zone test [13,32,33,34,36,37,38,39,40]. Other methods such as optical density [35,37,39], bacterial leakage [29], scanning electron microscopy [10,33], confocal laser scanning microscopy [13,21,27,30] and bacterial genomic profiling [21] were also used. Some bacterial activities and properties such as lactic acid production [21,27], metabolic activities [21,27,30], polysaccharides production [27], tolerance to acid and oxygen [27] and pH [10,27] were also assessed in a few of the included studies.

#### 3.3.5. Other Properties and Tests

Beside assessing the antibacterial effects, some studies investigated other properties such as fluoride release, degree of conversion, shear-bond strength, microhardness, compressive strength, tensile strength, flexural strength, depth of cure, softening, cytotoxicity and microleakage (Table 3).

#### 3.3.6. Control and Tested Groups

There was a variation in the materials used as control groups between the included studies (Table 3). Tested groups were either commercially available sealant materials or newly developed sealant materials.

### 3.4. Summary of Findings

Summaries of the findings are presented in Table 4 and Table 5.

Studies that used CFU counting to assess the antibacterial effects found a significant reduction in CFU counts with 5% ZnM [10], 2.5 and 5% SnM [10], 2.5 and 5% METAC [11], 5% DMAHDM [21,27], 2% TAT [12], 0.5 and 1% ZnO [28], 0.5 and 1% CaF_2_ [28], 4% MAE-DB [30], 2, 2.5, 3, 4 and 5% chitosan [31,34], 0.25, 0.5 and 1% Se [13], Teethmate F-1 [34], Seal & Protect [34] and 1% DMAE-CB [35] sealants in comparison to the controls or other tested groups (Table 4). There was a wide variation in the data reported among the included studies. The antibacterial effect of the antibacterial sealants was not significantly different after 2 weeks, 50 days, 2 months and 6 months aging in distilled water or phosphate-buffered saline [11,13,30,35,39].

Studies that assessed the antibacterial effects using an inhibition zone test reported the use of 1% CHX [32], 0.12% CHX [33], 0.25% Se [13], 2 and 2.5% chitosan [34], Teethmate F-1 [34,36,37,40], Seal & Protect [34], Clinpro [37] and Dryact Seal [39] sealants in comparison to the controls or other tested groups (Table 4).

The results of other studies that used optical density, bacterial leakage, scanning electron microscopy, confocal laser scanning microscopy and bacterial genomic profiling to report the antibacterial effects of the tested materials are reported in Table 4. Other bacterial activities such lactic acid production, metabolic activities, polysaccharide production and pH after exposure to various tested antibacterial resin-based sealants were reported in a few studies (Table 5).

## 4. Discussion

The incorporation of antibacterial agents into dental sealants has sought to develop resins with improved therapeutic properties to prevent dental caries. The present review mapped the studies that evaluated dental sealants containing antibacterial agents. This review’s findings evidenced how researchers have been assessing dental sealants and summarized the outcomes of the previous studies. Furthermore, this review summarized the main compounds that have been tested as antibacterial agents in dental sealants and evaluated the quality of the current evidence.

From the nineteen studies included in this systematic review, most of them (thirteen) showed a medium risk of bias. This finding was mainly based on the fact that the sample calculation and blinding were not included in all of them. It is common to observe that researchers consider previous studies to define the sample size, without critical thinking or indication of the sample calculation. This is a problem, especially when no statistically significant differences are detected in the test. The authors must be aware that a small sample size can decrease the statistical power and result in type II error. As the statistical power is not always reported, it is suggested that the sample size calculation and the power of the study are written in the manuscripts about this subject to improve the quality of research reports. Blinding is another parameter that is commonly not followed up [26]. The addition of antibacterial agents in dental resins frequently alters their physical properties, such as the color and viscosity of dental sealants. These characteristics can hinder the blinding process. As well as the reporting of sample size calculation, this parameter could assist in increasing the quality of studies.

The methods for analyzing the antibacterial activity of restorative materials have been criticized in the literature [26,41,42]. This stems from the lack of standardization of the tests, the misinterpretation of purely qualitative analyses or the misuse of quantitative tests. The methods addressed to analyze the dental sealants involved different outcomes. A meta-analysis was not performed due to the methodological variations, which should be more standardized to produce comparable data. Fortunately, all included studies used at least one quantitative test. In addition, the CFU counting test, which is the gold standard for evaluating microorganisms’ viability, was the most used. The inhibition zone test was the second most used. For some years now, some journals have been asking that authors not use this method.

A clear example of this was a publication by the Editorial Board of the Journal of Endodontics in 2007 [43]. The editors argued that this journal would no longer accept inhibition zone tests because antimicrobial agents can interact with the agar medium and remove ions from the environment. Furthermore, the agar medium’s buffering activity and the chemical interaction between the agar and the antibacterial agents can change the inhibition zone diameter and, consequently, the conclusions about the antibacterial effect. In addition, the zone of inhibition depends on the agent’s ability to permeate through the agar, which is essentially hydrophilic. Therefore, agents with greater hydrophobicity may induce a smaller inhibition zone, despite having a positive effect against the formation of biofilms. In conclusion, this is a test that provides uncertain information about the materials’ antimicrobial capacity, not necessarily reflecting the antimicrobial activity neither in vitro nor in vivo [43]. Therefore, we suggest that readers must have caution when reading and interpreting studies of resin-based sealants with antimicrobials that used only the zone of inhibition test to assess the materials’ antimicrobial activity.

Other methods such as scanning electron microscopy, confocal laser scanning microscopy, bacterial genomic profiling and tests to analyze the metabolic activity or the capacity to produce essential compounds for biofilm structure are interesting to improve the investigations’ quality findings. Few studies used a set of methods to analyze the dental sealants. However, as previously stated, most of them used CFU counting as the primary outcome. This method provides the most predictable effect because it does not depend solely on bacterial metabolic activity, such as the MTT test, or bacterial membrane integrity, such as microscopy with a live/dead kit [44]. CFU essentially relies on bacterial viability. Therefore, it is indicated that the CFU results are the main ones considered when analyzing the antimicrobial activity of dental materials with antimicrobial agents [44].

The other concern about the antimicrobial activity tests related to the characteristics of the microbial inoculum used [26,41,42]. From the nineteen studies selected, seventeen tested the dental sealants against *S. mutans*. This bacterium is directly related to the development of caries [45,46]. However, dental biofilm is much more complex, involving several microorganisms and microbial interactions, which increases the challenge for antimicrobial dental materials. Although some studies used other microorganisms, such as *L. acidophilus, S. oralis, C. albicans, S. salivarius* and *S. sobrinus*, only one study considered the complexity of dental biofilm [21]. This study used multispecies biofilms cultured from pooled saliva of high-caries risk and low-caries risk pediatric dental patients. Currently, we need better in vitro studies that use biofilms with a longer maturation time and microorganisms directly associated with dental caries. The use of biofilms cultured from pooled human saliva seems to be the best alternative for in vitro evaluations to better predict in vivo outcomes. Pooled saliva is recommended to be used to reduce the variation in thickness and densities of biofilms between individuals [47,48].

Among the antibacterial agents/compounds evaluated, those based on quaternary ammonium compounds stand out. This finding is similar to the previous one in the scoping review about antimicrobial agents in restorative resin-based materials, when many studies have tested adhesive systems containing methacryloyloxidodecylpyridinium bromide (MDPB) [26]. Another material frequently found was that composed of fluoride. Fluoride can reduce the metabolic activity of microorganisms or induce the formation of fluorapatite after dental demineralization occurs [49]. Fluorapatite is more resistant to demineralization than hydroxyapatite, making the tooth less susceptible to demineralization when fluoride is present in the environment [49]. However, the study with the highest level of evidence on resinous materials with fluoride shows that other antimicrobial agents, such as MDPB, induce a better antimicrobial effect than materials containing only fluoride [50]. This finding must occur due to the low fluoride concentration capable of being incorporated into resinous materials. With regard to dental sealant types, there is insufficient evidence about the effectiveness of sealants with fluorine compared to other types of sealants [7].

In addition to antimicrobial analyses, many studies have evaluated the physicochemical properties by incorporating antimicrobial agents. Since dental sealants must remain in function, with adhesion to the tooth, and without suffering fractures, the authors must analyze the materials’ physicochemical properties. A material that rapidly suffers degradation or exhibits mechanical properties that lead to a loss of function will likely need to be replaced or repaired. The degree of conversion, which analyzes the carbon-carbon double bonds’ capacity to convert into carbon-carbon single bonds [51], was the most commonly used test among the studies.

This chemical property is frequently altered by incorporating antimicrobial agents and bioactive charges due to the difference in the refractive index between the resin matrix and the added compounds [52,53]. The viscosity of resins after incorporating agents can also be modified, changing the degree of conversion [54] and the adhesion to dental tissues, which is the primary outcome to be assessed. The studies that tested the degree of conversion showed promising results with the incorporation of antimicrobial agents such as ZnM, SnM, DMAHDM, TAT, chitosan and DMAE-CB. The same effect was also found when the adhesion to the tooth structure was tested, such as the shear-bond strength. These findings may be due to the low concentration of agents incorporated in the sealants, which was up to 5 wt % for most studies. Overall, acceptable physicochemical and antimicrobial properties were found for the sealants with most of the studied antibacterial agents.

There is a trend toward formulating and evaluating dental resin-based sealants with antibacterial agents. Even though there is no standardization among the studies, and most of the studies showed issues related to the risk of bias, most of the studies used the gold-standard test to evaluate the antimicrobial activity, which is a very positive finding. The improving of inoculum source to produce a high challenge scenario for the novel dental sealants should be a fundamental goal of the future in vitro studies. The use of more complex methods to better predict antimicrobial results may be the way to accelerate the translation of knowledge from the bench to the clinic.

## 5. Conclusions

In summary, based on the included in vitro studies, the addition of antibacterial agents in resin-based dental sealants could have promising antimicrobial effects. These effects may improve the function of sealants as materials for caries prevention and improve their therapeutic activity. However, standardization of the in vitro studies’ protocols and in situ studies and clinical trials to assess these effects and support the findings are recommended.

## Figures and Tables

**Table 1:** Search strategies.

**Database: PubMed**	
#1	(“fluoride” [tiab] OR “calcium” [tiab] OR “hydroxyapatite” [tiab] OR “remineral*” [tiab] OR “Preven*” [tiab] OR “Antimicrobial” [tiab] OR “antibacterial” [tiab] OR “biofilm” [tiab] OR “bioactiv*” [tiab])
#2	(“Sealant*” [tiab] OR “Sealing*” [tiab] OR “Sealer” [tiab] OR “pit and fissure sealants” [Mesh])
#3	#1 and #2
**Database: Web of Knowledge**	
#1	(antibacterial OR antimicrobial OR remineral* OR demineral* OR hydroxyapatite OR calcium* OR fluorid* OR bioactiv* OR Biofilm)
#2	(sealant* OR sealing* OR (pit AND fissure))
#3	#1 and #2
**Database: SCOPUS**	
#1	(TITLE-ABS-KEY (sealant* OR sealing* OR (pit AND fissure)))
#2	(TITLE-ABS-KEY (antibacterial OR antimicrobial OR remineral* OR hydroxyapatite OR calcium* OR fluorid* OR bioactiv* OR biofilm*))
#3	#1 and #2
**Database: OVID (Ovid MEDLINE(R) and Epub Ahead of Print, In-Process & Other Non-Indexed Citations, Daily and Versions(R) 1946 to 1 June 2020)**	
#1	(sealant or sealing or (pit and fissure sealant)).af
#2	(fluoride or calcium or hydroxyapatite or remineral* or antimicrobial or antibacterial or prevent or biofilm or bioactiv*).af.
#3	#1 and #2

**Table 2:** Risk of bias appraisal.

Study	Sampling Bias		Assessment Bias			Reporting Bias		Funding Bias	Conflict Bias	Risk of Bias
	Sample Size Calculation	Sample Preparation	Blinding	Assessment Methods	Presence of Control Group	Definitive Values	Quantitative Analysis			
Coco et al., 2020 [10]	+	−	+	−	−	+	−	−	−	Low
Garcia et al., 2020 [11]	+	−	+	+	−	−	−	−	−	Low
Ibrahim et al., 2020 [21]	+	−	+	−	−	+	−	−	+	Medium
Monteiro et al., 2020 [12]	+	−	+	+	−	−	−	−	−	Low
Ibrahim et al., 2019 [27]	+	−	+	−	−	+	−	−	+	Medium
Swetha et al., 2019 [28]	+	−	+	+	−	−	−	−	−	Low
Zmener et al., 2019 [29]	+	−	+	+	−	−	−	−	+	Medium
Yu et al., 2016 [30]	+	−	+	−	−	+	−	−	+	Medium
Rajabnia et al., 2016 [31]	+	−	+	+	−	−	−	−	−	Low
Shanmugaavel et al., 2015 [32]	+	−	+	+	−	−	−	−	−	Low
Hamilton et al., 2014 [33]	+	−	+	+	−	+	−	+	+	Medium
Tran et al., 2013 [13]	+	−	+	−	−	+	−	−	+	Medium
Mahapoka et al., 2012 [34]	+	−	+	−	−	−	−	−	+	Medium
Feng Li et al., 2011 [35]	+	−	+	−	−	+	−	−	+	Medium
Kumar et al., 2010 [36]	+	−	+	+	+	−	−	+	+	Medium
Naorungroj et al., 2010 [37]	+	−	+	−	+ *	−	−	+	+	Medium
Menon et al., 2007 [38]	+	−	+	+	−	−	−	+	+	Medium
Matalon et al., 2003 [39]	+	−	+	−	+ *	−	−	+	+	Medium
Loyola-Rodriguez et al., 1996 [40]	+	−	+	+	+ **	−	−	+	+	Medium

(+) Yes; (−) no; (*) they used only blank well/disc.; (**) they used commercially available sealant but not the same resin matrix as the experimental group.

**Table 3:** Characteristics of included studies.

Study	Group Sample Size	Sample Type or Measurements	Light Curing Time	Assessment Methods	Bacteria	Antibacterial Agents	Other Tests/Properties Assessed	Control Groups	Tested Groups
Cocco et al., 2020 [10]	4	Sealants were applied and polymerized on HA discs (1.25 cm in diameter)	-	CFU SEM pH	*S. mutans* *S. oralis* *C. albicans*	Zinc methacrylate (ZnM) di-nbutyldimethacrylate-tin (SnM)	DC Translucency parameter microshear bond strength FS Depth of cure Cytotoxicity assay	Resin Base = TEGDMA + BisGMA + glycerol dimethacrylate phosphate + Water +, phenylbis (2,4,6-tri-methylbenzoyl)-phosphine oxide + diphenyliodo- nium hexafluorophosphate + nanometric silica	Resin Base + 2.5% ZnM Resin Base + 5% ZnM Resin Base + 2.5% SnM Resin Base + 5% SnM
Garcia et al., 2020 [11]	3	4 mm diameter × 1 mm thickness	30 s on each side	CFU (with or without aging for 50 days in distilled water)	*S. mutans*	[2(methacryloyloxy)ethyl] trimethylammonium chloride (METAC)	DC Softening in solvent Ultimate tensile strength Contact angle and SFE microshear bond strength and cytotoxicity evaluation (with and without aging for 50 days in distilled water at 37 °C.)	60 wt % BisGMA + 40 wt % TEGDMA + 1 mol % CQ + 1 mol % 4E + 0 wt % METAC	60 wt % BisGMA + 40 wt % TEGDMA + 1 mol % CQ + 1 mol % 4E+ 2.5 wt % METAC 60 wt % BisGMA + 40 wt % TEGDMA + 1 mol % CQ + 1 mol % 4E + 5 wt % METAC
Ibrahim et al., 2020 [21]	Genomic profiling = 3 CLSM = 2 6 × 3 repetition (all other)	9 mm diameter × 2 mm thickness	60 s on each side	CFU Metabolic Activity (MTT) CLSM Lactic Acid Production Genomic Profiling of Saliva-Derived Biofilms	Pooled saliva from healthy high and low caries-risk pediatric patients	Dimethylaminohexadecyl methacrylate (DMAHDM)	-	50% PEHB (44.5% PMGDM + 39.5% EBPADMA + 10% HEMA + 5% BisGMA + 1% BAPO) + 50% Glass	45% PEHB + 50% Glass + 0% NACP + 5% DMAHDM 45% PEHB + 30% Glass + 20% NACP + 5% DMAHDM
Monteiro et al., 2020 [12]	3	4 mm diameter × 1 mm thickness	30 s on each side	CFU	*S. mutans*	TAT	DC Softening Ultimate tensile strength (UTS) Contact angle and SFE	Resin Base = 50 wt % BisGMA + 50 wt % TEGDMA + 1 mol % CQ+4E + BHT 0.01% + Calcium Tungstate 30 wt % + 0.7 wt % Colloidal silica	Resin Base + 2 wt % α-TCP + 2 wt % TAT
Ibrahim et al., 2019 [27]	6 × 3 repetition	9 mm diameter × 2 mm thickness	60 s on each side	CFU Metabolic Activity (MTT) Polysaccharide Production CLSM Acid-Neutralizing Activity Lactic Acid Production	*S. mutans*	Dimethylaminohexdecyl methacrylate (DMAHDM)	-	Virtuoso Flowable Composite 50% PEHB (44.5% PMGDM + 39.5% EBPADMA + 10% HEMA + 5% BisGMA + 1% BAPO) + 50%Glass	45% PEHB + 50% Glass + 0% NACP + 5% DMAHDM 50% PEHB + 30% Glass + 20% NACP + 0% DMAHDM 45% PEHB + 30% Glass + 20% NACP + 5% DMAHDM
Swetha et al., 2019 [28]	7	Coating equal amount of sealant material on to the walls of eppendorf tubes	40 s (7 cycles from the top to the bottom of the tube)	Direct Contact Test (CFU)	*S. mutans* *L. acidophilus*	Zinc Oxide (ZnO) and Calcium Fluoride (CaF_2_) nanoparticles (NPs)	compressive and flexural strengths	Plain fissure sealant (PFS)	PFS + 0.5 or 1 wt % ZnO PFS + 0.5 or 1 wt % CaF_2_ PFS + 0.5 or 1 wt % ZnO + 0.5 or 1 wt % CaF_2_
Zmener et al., 2019 [29]	10	Randomly assigned cleaned, sterilized, non-carries extracted human third molars without overfilling the pit and fissures	20 s	Bacterial Leakage Testing (frequencies and median survival time)	*Enterococcus faecalis*	Modified calcium phosphate (MCP)	-	Embrace Wet Bonda (EWB) A commercially available P&F sealant Clinpro (CLPR)	EWB + MCP (EWBMCP)
Yu et al., 2016 [30]	Metabolic Activity = 6 CFU = 5	8 mm diameter	20 s	CFU Metabolic Activity (Cell Counting Kit-8) CLSM (All the tests with or without aging 6 months in distilled water)	*S. mutans*	2-methacryloxylethyl dodecyl methyl ammonium bromide (MAE-DB)	-	Eco-S Sealant Clinpro™ Sealant	Eco-S Sealant + 4 wt % MAE-DB
Rajabnia et al., 2016 [31]	3	200 μL of each sealant group was poured into 0.5 mL microtubes	40 s	CFU	*S. mutans*	Chitosan	-	Clinpro + 0 wt % Chitosan	Clinpro + 1, 2, 3, 4 or 5 wt % Chitosan
Shanmugaavel et al., 2015 [32]	5	6 mm diameter	-	Inhibition Zone	*S. mutans* *L. acidophilus*	20% chlorhexidine digluconate liquid (CHX)	Compressive strength (CS) diametrical tensile strength	Conventional glass ionomer sealants (GIS) (Fuji VII) Clinpro	GIS + 1% CHX Clinpro + 1% CHX
Hamilton et al., 2014 [33]	10	5 mm diameter × 2 mm thickness	40 s	Inhibition Zone	*S. mutans*	Electrospun nylon-6 (N6) + Chitosan (CH)	Flexural strength Vickers microhardness	Helioseal Clear 0.12% Chlorhexidine (CHX) solution	Resin Base = 60% Bis-GMA + 40% TEGDMA + 0.5% CQ + 1% (Dimethylamino)ethyl methacrylate (DMAEMA) Resin Base + 1, 2.5 or 5 wt % N6 Resin Base + 1, 2.5 or 5 wt % CH
Tran et al., 2013 [13]	6	7 mm discs	-	CFU Inhibition Zone (above tests with or without aging 2 months in PBS) CLSM	*S. mutans* *S. salivarius*	Organo-selenium	-	Selenium-free sealant (BisGMA + TEGDMA + multifunctional monomer for methacrylate formation + CQ)	0.1%, 0.2%, 0.25%, 0.5% or 1% Selenium-containing dental sealants (SeLECT-DefenseTM sealant)
Mahapoka et al., 2012 [34]	3	5 mm diameter × 2 mm thickness	40 s	Inhibition Zone CFU	*S. mutans*	Freeze-dried chitosan Whiskers	DC Vickers hardness Depth of cure	Resin Base = 57 wt % Bis-GMA + 41.9 wt % TEGDMA + 0.86 wt % 2-dimethylaminoethyl methacrylate + 0.24 wt % CQ Delton Teethmate™F-1 Seal&Protect™	Resin Base + 1 wt % or 1.5 wt % or 2 wt % or 2.5 wt % Chitosan
Feng Li et al., 2011 [35]	5	8 mm diameter	20 s	CFU (with or without aging for 6 months in distilled water) OD	*S. mutans*	Methacryloxylethyl cetyl dimethyl ammonium chloride (DMAE-CB)	Contact angles Vickers microhardness DC Microleakage	Helioseal Helioseal F	Helioseal + 1 w% DMAE-CB
Kumar et al., 2010 [36]	10	5 mm diameter × 3 mm thickness	-	Inhibition Zone	*S. mutans*	Fluoride	-	-	Glass ionomer cement: Fuji IX GP Ketac molar Pit and fissure sealants: Teethmate-F1 Helioseal-F
Naorungroj et al., 2010 [37]	inhibition zone = 4 OD = 9	6-mm sterile paper disks 6 mm diameter × 2 mm thickness obtained from labial surface of lower anterior bovine teeth	20 s	Inhibition Zone (paper disk) Inhibition Zone (enamel disk) OD	*S. mutans* *L. acidophilus*	Fluoride	-	Blank disks	Clinpro Embrace WetBond UltraSeal XT plus
Menon et al., 2007 [38]	15	5 mm diameter	20 s	Inhibition Zone	*S. mutans* *L. acidophilus*	Fluoride	-	Helioseal -	Teethmate-F1 Helioseal-F
Matalon et al., 2003 [39]	8	4 mm diameter	-	Inhibition Zone OD (with or without aging for 2 weeks and 1 month in PBS)	*S. mutans*	Fluoride	-	Blank wells	Helioseal F Ultraseal XT Conseal F Dyract Seal
Loyola-Rodriguez et al., 1996 [40]	3	3 mm diameter	-	Inhibition Zone	*S. mutans* MT8148, NG71 and GS5 (serotype c); *S. mutans* MT703R (serotype e); *S. mutans* OMZ175 (serotype f). *S. sobrinus* MT4532, MT6223 and 6715 (serotype g).	Fluoride	Fluoride release test	Helioseal	FluoroShieldTM Teethmate-FTM

HA: hydroxyapatite, CFU: colony forming unit, SEM: scanning electron microscopy, *S. mutans: Streptococcus mutans*, *S. oralis: Streptococcus oralis*, *C. albicans: Candida albicans*, DC: degree of conversion, FS: flexural strength, TEGDMA: triethylene glycol dimethacrylate, BisGMA: bisphenol A-glycidyl methacrylate, SFE: surface free energy, CQ: Ccamphorquinone, 4E: ethyl 4-dimethylaminobenzoate, CLSM: confocal laser scanning microscopy, MTT: (3-(4,5-dimethylthiazol-2-yl)-2,5-diphenyltetrazolium bromide) tetrazolium reduction assay, PMGDM: pyromellitic glycerol dimethacrylate, EBPADMA: ethoxylated bisphenol A dimethacrylate, HEMA: 2-hydroxyethyl methacrylate, BAPO: phenyl-bis(2,4,6-trimethylbenzoyl)-phosphine oxide, NACP: nanoparticles of amorphous calcium phosphate, TAT: 1,3,5-tri acryloyl hexahydro-1,3,5-triazine, α-TCP: α-tricalcium phosphate, BHT: butylated hydroxytoluene, OD: optical density, *L. acidophilus: Lactobacillus acidophilus*, PBS: phosphate-buffered saline, Organo-selenium: organo-selenium (3-[3-((2-[22]-ethyldiselenyl))-propionyloxy]-butyric acid 2-(2-methyl-acryloyloxy)-ethyl ester).

**Table 4:** Antibacterial effects of dental sealants using CFU counting, inhibition zone, optical density, bacterial leakage, genomic profiling, confocal laser scanning microscopy and scanning electron microscopy.

Antibacterial Effect				
Assessment Method	Study	Intervention (Mean ± SD)	Control (Mean ± SD)	Summary of Results
**CFU Counting**	Cocco et al., 2020 [10]	-	-	*Streptococcus mutans*: CFU count for the sealant containing ZnM 5% showed significant reduction (40%) in comparison to control groups, while ZnM 2.5% did not show significant difference. Further reductions in the CFU of *S. mutans* were observed from both SnM 2.5% and 5% surfaces (70%) in comparison to control groups (*p* > 0.05).
				*S. oralis* and *C. albicans*: CFU count for the sealant containing SnM 5% showed a significant reduction in comparison to control groups (*p* > 0.05).
	Garcia et al., 2020 [11]	**Biofilm formation (log CFU/mL):**	**Biofilm formation (log CFU/mL):**	There was significant difference between 2.5% or 5% METAC in comparison to control groups in immediate and long-term CFU count of the form biofilm and planktonic bacteria. There was no significant difference between the immediate and long-term CFU count for each group.
		**Immediate:**	**Immediate:**	
		2.5% METAC 5.05 (± 0.13)	0% METAC 6.31 (± 0.10)	
		5% METAC 4.93 (± 0.25)	Negative control (-)	
		**Long-term:**	**Long-term:**	
		2.5% METAC 4.98 (± 0.23)	0% METAC 6.26 (± 0.19)	
		5% METAC 5.02 (± 0.13)	Negative control (-)	
		**Planktonic bacteria:**	**Planktonic bacteria:**	
		**Immediate:**	**Immediate:**	
		2.5% METAC 8.02 (± 0.14)	0% METAC 9.00 (± 0.17)	
		5% METAC 7.92 (± 0.21)	Negative Control 9.03 (± 0.06)	
		**Long-term:**	**Long-term:**	
		2.5% METAC 7.95 (± 0.27)	0% METAC 9.05 (± 0.24)	
		5% METAC 7.86 (± 0.15)	Negative control 9.03 (± 0.06)	
	Ibrahim et al., 2020 [21]	-	-	Overall, the sealants containing 5% DMAHDM + 0% NACP showed significant reductions in CFU count for total microorganisms, total streptococci, lactobacilli and mutans streptococci in saliva-drived biofilm from both high and low caries-risk pediatric patients in comparison to the control (*p* < 0.05). However, the sealant containing DMAHDM + NACP showed less reduction in comparison to the sealant containing only DMAHDM (*p* < 0.05).
	Monteiro et al., 2020 [12]	**Biofilm (log CFU/mL)**	**Biofilm (log CFU/mL)**	The sealant containing 2 wt % α-TCP + 2 wt % TAT showed a significant reduction in CFU counts in comparison to the control group (*p* < 0.05).
		2 wt % α-TCP + 2 wt % TAT (4.95 ± 0.30)	0 wt % α-TCP + 0 wt % TAT (6.38 ± 0.57)	
		Negative control (-)	Negative control (-)	
		**Planktonic Bacteria (log CFU/mL)**	**Planktonic Bacteria (log CFU/mL)**	
		2 wt % α-TCP + 2 wt % TAT (7.73 ± 0.56)	0 wt % α-TCP + 0 wt % TAT (9.21 ± 0.14)	
		Negative control (-)	Negative control (9.14 ± 0.10)	
	Ibrahim et al., 2019 [27]	-	-	The sealants containing 5% DMAHDM with or without NACP showed significant reductions in CFU count in comparison to the other sealants (*p* < 0.05).
	Swetha et al., 2019 [28]	**PFS + 0.5 wt % ZnO**	**Plain PFSs (Control)** *S. mutans* (129.29 ± 26.552) *L. acidophilus* (53.07 ± 7.829)	CFU count of all experimental sealants showed statistically significant difference in comparison to control group (*p* < 0.001)
		*S. mutans* (8.71 ± 5.894)		
		*L. acidophilus* (7.64 ± 1.909)		
		**PFS + 0.5 wt % CaF_2_**		
		*S. mutans* (12.21 ± 2.612)		
		*L. acidophilus* (8.50 ± 4.223)		
		**PFS + 0.5 wt % ZnO + 0.5 wt % CaF_2_**		
		*S. mutans* (1.50 ± 1.190)		
		*L. acidophilus* (2.43 ± 0.673)		
		**PFS + 1 wt % ZnO**		
		*S. mutans* (0.93 ± 0.976)		
		*L. acidophilus* (3.21 ± 1.113)		
		**PFS + 1 wt % CaF_2_**		
		*S. mutans* (5.07 ± 2.244)		
		*L. acidophilus* (2.93 ± 0.886)		
		**PFS + 1 wt % ZnO + 1 wt % CaF_2_**		
		*S. mutans* (0.57 ± 0.450)		
		*L. acidophilus* (0.64 ± 0.690)		
	Yu et al., 2016 [30]	**Colony-forming units (CFU) counts from *S. mutans* biofilms on the material surfaces:**	**Clinpro™ Sealant**	The sealant containing 4% MAE-DB showed a significant reduction in CFU count in comparison to the controls (*p* < 0.05).
		Without aging (-)	Without aging (6.09 ± 0.54) × 108	
		With aging (-)	With aging (5.8 ± 0.66) × 108	
		**Eco-S Sealant + 4 wt % MAE-DB**	**Eco-S Sealant**	
		Without aging (4.74 ± 0.97) × 106	Without aging (6.43 ± 0.75) × 108	
		With aging (4.83 ± 1.16) × 106	With aging (6.25 ± 0.66) × 108	
		**Colony forming units (CFU) counts from *S. mutans* biofilms in the material eluents:**	**Clinpro™ Sealant**	The sealant containing 4% MAE-DB showed no significant reduction in CFU count in comparison to the controls.
		Without aging (-)	Without aging (6.26 ± 0.46) × 108	
		With aging (-)	With aging (6.55 ± 0.44) ×108	
		**Eco-S Sealant + 4 wt % MAE-DB**	**Eco-S Sealant**	
		Without aging (6.45 ± 0.61) × 108	Without aging (6.79 ± 0.7) × 108	
		With aging (6.62 ± 0.47) × 108	With aging (6.84 ± 0.53) × 108	
	Rajabnia et al., 2016 [31]	**(CFU/mL)**	**(CFU/mL)** 0 wt % chitosan(-)	The sealants containing 2, 3, 4 and 5% of CH showed a significant reduction in CFU count in 1 month in comparison to the control and 1% CH groups (*p* < 0.001). In general, there were significant differences between the groups (*p* < 0.001).
		**24 h**		
		1 wt % chitosan (-)		
		2 wt % chitosan 2443.33 ± 51.316		
		3 wt % chitosan 1440.00 ± 36.056		
		4 wt % chitosan 871.67 ± 12.583		
		5 wt % chitosan 599.33 ± 9.018		
		**48 h**		
		1 wt % chitosan (-)		
		2 wt % chitosan 2523.33 ± 68.069		
		3 wt % chitosan 1413.33 ± 32.146		
		4 wt % chitosan 836.33 ± 6.506		
		5 wt % chitosan 563.67 ± 12.342		
		**3 months**		
		1 wt % chitosan (-)		
		2 wt % chitosan 2020.67 ± 20.33		
		3 wt % chitosan 1373.33 ± 25.166		
		4 wt % chitosan 782.00 ± 33.956		
		5 wt % chitosan 361.67 ± 17.559		
		Note: other timepoints were measured but not reported here.		
	Tran et al., 2013 [13]	**CFU/sealant disc for *S. salivarius***	**CFU/sealant disc for *S. salivarius***	The 1% Se containing sealant completely inhibited the growth of *S. salivarius* (*p* < 0.05) in comparison to the other groups. The 0.25%, 0.5% and 1% Se containing sealants completely inhibited the growth of *S. mutans* (*p* < 0.05) in comparison to the other groups, and 2 months aging in PBS 0.5% and 1% Se containing sealants completely inhibited the growth of *S. mutans* (*p* < 0.05) in comparison to the other groups.
		0.1% selenium (-)	selenium-free sealant 4 × 104	
		1% selenium 0		
		**CFU/sealant disc for *S. mutans***	**CFU/sealant disc for *S. mutans***	
		0.2% selenium (-)	selenium-free sealant 2 × 105	
		0.25% selenium 0		
		0.5% selenium 0		
		1% selenium 0		
		**CFU/sealant disc for *S. mutans* after 2 months aging**	**CFU/sealant disc for *S. mutans* after 2 months aging**	
		0.2% selenium -	0.2% selenium -	
		0.5% selenium 0	0.5% selenium -	
		1% selenium 0	1% selenium -	
	Mahapoka et al., 2012 [34]	**Bacterial reduction (%)**	**Bacterial reduction (%)**	The 2%, 2.5% chitosan, Teethmate™ F-1 and Seal&Protect™ sealants showed a significantly higher bacterial reeducation rate in comparison to the other groups (*p* < 0.05). Seal&Protect showed the highest bacterial reeducation rate.
		**(BRR) = [(N1 − N2)/N1] × 100**	**(BRR) = [(N1 − N2)/N1] × 100**	
		**Where N1 and N2 = viable count at 0 and 12 h.**	**Where N1 and N2 = viable count at 0 and 12 h.**	
		1% chitosan 31.2 (4.7)	Resin base 13.7 (2.9)	
		1.5% chitosan 39.2 (3.7)	Delton^®^ 25.9 (3.8)	
		2% chitosan 72.2 (0.6)	Seal&Protect™ 83.1 (0.7)	
		2.5% chitosan 75.9 (0.6)	Teethmate™ F-1 76.9 (0.3)	
	Feng Li et al., 2011 [35]	-	-	The 1% DMAE-CB sealant showed a significant reduction in CFU count in comparison to the controls with or without aging (*p* < 0.05). There was no significant difference between the aged and non-aged samples in each group (*p* > 0.05).
**Inhibition Zone**	Shanmugaavel et al., 2015 [32]	**Inhibition zone (mm)**	**Inhibition zone (mm)**	The sealants containing 1% CHX showed significant increase in the inhibition zones against *S. mutans* and *L. acidophilus* at 0 day in comparison to the controls. These differences were still observed after 7 and 30 days but less pronounced (*p* < 0.05).
		***S. mutans***	***S. mutans***	
		**0 day**	**0 day**	
		GIS + 1% CHX 10.36 mm	GIS 7.28 mm	
		Clinpro + 1% CHX 14.82	Clinpro 11.96 mm	
		**7 days**	**7 days**	
		GIS + 1% CHX 5.7 mm	GIS 0 mm	
		Clinpro + 1% CHX 8.68	Clinpro 0 mm	
		**30 days**	**30 days**	
		GIS + 1% CHX 1.67 mm	GIS 0 mm	
		Clinpro + 1% CHX 5.83 mm	Clinpro 0 mm	
		***L. acidophilus***	***L. acidophilus***	
		**0 day**	**0 day**	
		GIS + 1% CHX 9.7 mm	GIS 4.16 mm	
		Clinpro + 1% CHX 10.18 mm	Clinpro 4.4 mm	
		**7 days**	**7 days**	
		GIS + 1% CHX 5.02 mm	GIS 0 mm	
		Clinpro + 1% CHX 8.16 mm	Clinpro 0 mm	
		**30 days**	**30 days**	
		GIS + 1% CHX 1.5 mm	GIS 0 mm	
		Clinpro + 1% CHX 4.83 mm	Clinpro 0 mm	
	Hamilton et al., 2014 [33]	-	-	The 0.12% CHX solution was the only group that showed an inhibition zone (4 mm) for S. mutans at 24, 48 and 120 h. There was no inhibition zone for all experimental groups. Only the positive control, that is, chlorhexidine 0.12% solution demonstrated an inhibition zone (4 mm) against S. mutans during the time of the study (24, 48 and 120 h). No inhibition zone was observed in any of the experimental groups tested (data not shown).
	Tran et al., 2013 [13]	-	-	The 0.25% containing Se sealant completely inhibited the growth of s. mutans in comparison to the control.
	Mahapoka et al., 2012 [34]	**Width of inhibition zone (mm)**	**Width of inhibition zone (mm)**	The 2%, 2.5% chitosan, Teethmate™ F-1 and Seal&Protect™ sealants showed a higher inhibition zone in comparison to the other groups. Seal&Protect showed the highest bacterial reeducation rate.
		2.5% chitosan whisker (10.7 ± 0.3)	Control (5.0 ± 0)	
		2% chitosan whisker (10.1 ± 0.2)	Delton^®^ (5.0 ± 0)	
		1.5% chitosan whisker (5.0 ± 0)	Seal&Protect™ (15.2 ± 0.2)	
		1% chitosan whisker (5.0 ± 0)	Teethmate™ F-1 (11.4 ± 0.2)	
	Kumar et al., 2010 [36]	**Width of inhibition zone (mm)** Ketac molar (2.18 ± 0.24) Fuji IX GP (5.50 ± 0.62) Teethmate-F1 (8.43 ± 0.42) Helioseal-F (0.00 ± 0.00)	-	The Teethmate-F1 sealant showed the largest inhibition zone while the Helioseal-F sealant showed no inhibition zone (*p* = 00). Seal&Protect showed the highest inhibition zone.
	Naorungroj et al., 2010 [37]	**Inhibition zone in mm (paper disk) *L. acidophilus***	**Inhibition zone in mm (paper disk) *L. acidophilus* and *S. mutans*** Control (0.0 ± 0.0)	The Clinpro sealant showed the largest inhibition zone against L. acidophilus. The Embrace sealant showed an inhibition zone against S. mutans using both paper and enamel disk (note: no *p*-value was given).
		Clinpro (17.6 ± 2.8)		
		Embrace (6.0 ± 0.0)		
		UltraSeal (6.0 ± 0.0)		
		***S. mutans***		
		Clinpro (6.8 ± 0.5)		
		Embrace (7.9 ± 1.3)		
		UltraSeal (6.0 ± 0.0)		
		**Inhibition zone in mm (enamel disk) *L. acidophilus***	**Inhibition zone in mm (enamel disk) *L. acidophilus* and *S. mutans*** Control (0.0 ± 0.0)	
		Clinpro (9.8 ± 0.3)		
		Embrace < 6.0		
		UltraSeal (6.0 ± 0.0)		
		***S. mutans***		
		Clinpro (6.0 ± 0.0)		
		Embrace (6.5 ± 0.0)		
	Menon et al., 2007 [38]	**Inhibition zone in mm**	**Inhibition zone in mm**	The Teethmate-F1 showed a significant difference in the inhibition zone between *S. mutans* and *L. acidophilus*.
		***S. mutans***	***S. mutans***	
		Teethmate-F1 (11.763 ± 0.391)	Helioseal 0	
		Helioseal-F 0	-	
		***L. acidophilus***	***L. acidophilus***	
		Teethmate-F1 (13.583 ± 0.318)	Helioseal 0	
		Helioseal-F 0	-	
	Matalon et al., 2003 [30]	**Inhibition zone in mm**	-	The Dyract Seal sealant showed an inhibition zone while the other sealants did not show any inhibition zone.
		Conseal F (0 ± 0)		
		Helioseal F (0 ± 0)		
		Ultraseal XT (0 ± 0)		
		Dyract Seal (6.62 ± 0.51)		
	Loyola-Rodriguez et al., 1996 [40]	**Width of inhibition zone (mm)**	**Width of inhibition zone (mm)**	The Teethmate-F sealant was the only material that showed an inhibition zone against all strains of *S. mutans.*
		***S. mutans***	***S. mutans***	
		**MT8148**	**MT8148**	
		Teethmate-FTM (1.0 ± 0.0)	Helioseal (0 ± 0)	
		FluoroShieldTM (0 ± 0)	-	
		**NG71**	**NG71**	
		Teethmate-FTM (1.0 ± 0.0)	Helioseal (0 ± 0)	
		FluoroShieldTM (0 ± 0)	-	
		**GS5**	**GS5**	
		Teethmate-FTM (1.0 ± 0.0)	Helioseal (0 ± 0)	
		FluoroShieldTM (0 ± 0)		
		**MT703R**	**MT703R**	
		Teethmate-FTM (0.6 ± 0.3)	Helioseal (0 ± 0)	
		FluoroShieldTM (0 ± 0)	-	
		**OMZ175**	**OMZ175**	
		Teethmate-FTM (0.6 ± 0.3)	Helioseal (0 ± 0)	
		FluoroShieldTM (0 ± 0)	-	
		***S. sobrinus***	***S. sobrinus***	
		**6715**	**6715**	
		Teethmate-FTM (1.0 ± 0.3)	Helioseal (0 ± 0)	
		FluoroShieldTM (0 ± 0)	-	
		**MT4532**	**MT4532**	
		Teethmate-FTM (0.6 ± 0.3)	Helioseal (0 ± 0)	
		FluoroShieldTM (0 ± 0)	-	
		**MT6223**	**MT6223**	
		Teethmate-FTM (1.0 ± 0.0)	Helioseal (0 ± 0)	
		FluoroShieldTM (0 ± 0)	-	
**Optical Density**	Feng Li et al., 2011 [35]	-	-	There were no significant differences between the groups (*p* > 0.05).
	Naorungroj et al., 2010 [37]	***L. acidophilus* suspensions exposed to pit and fissure sealants.**	***L. acidophilus* suspensions exposed to pit and fissure sealants**	No statistical analysis was mentioned in this study.
		**No wash**	**No wash**	
		Clinpro (0.075 ± 0.010)	**Control (0.455 ± 0.019)**	
		Embrace (0.140 ± 0.029)	**-**	
		UltraSeal (0.056 ± 0.002)	**-**	
		**30-min wash**	**30-min wash**	
		Clinpro (0.075 ± 0.005)	**Control (0.431 ± 0.014)**	
		Embrace (0.086 ± 0.005)	**-**	
		UltraSeal (0.086 ± 0.003)	**-**	
		**24-h wash**	**24-h wash**	
		Clinpro (0.077 ± 0.003)	**Control (0.429 ± 0.017)**	
		Embrace (0.098 ± 0.029)	**-**	
		UltraSeal (0.067 ± 0.005)	**-**	
		**48-h wash**	**48-h wash**	
		Clinpro (0.103 ± 0.026)	**Control (0.405 ± 0.012)**	
		Embrace (0.098 ± 0.065)	**-**	
		UltraSeal (0.106 ± 0.026)	**-**	
		***S. mutans* suspensions exposed to pit and fissure sealants.**	***S. mutans* suspensions exposed to pit and fissure sealants**	
		**No wash**	**No wash**	
		Clinpro (0.068 ± 0.007)	**Control (0.441 ± 0.024)**	
		Embrace (0.117 ± 0.018)	**-**	
		UltraSeal (0.051 ± 0.002)	**-**	
		**30-min wash**	**30-min wash**	
		Clinpro (0.073 ± 0.005)	**Control (0.557 ± 0.060)**	
		Embrace (0.088 ± 0.008)	**-**	
		UltraSeal (0.066 ± 0.001)	**-**	
		**24-h wash**	**24-h wash**	
		Clinpro (0.053 ± 0.003)	**Control (0.423 ± 0.019)**	
		Embrace (0.054 ± 0.003)	**-**	
		UltraSeal (0.341 ± 0.044)	**-**	
		**48-h wash**	**48-h wash**	
		Clinpro (0.113 ± 0.028)	**Control (0.398 ± 0.021)**	
		Embrace (0.054 ± 0.004)	-	
		UltraSeal (0.433 ± 0.026)	*-*	
	Matalon et al., 2003 [39]	**Fresh material**	**Fresh material**	The Dyract Seal showed the highest antibacterial affect in comparison to other groups; this difference was significant at 0 timepoint and for 2-week aged samples but not the 1-month aged samples (*p* < 0.0001).
		Conseal F (2.659 ± 0.401)	Control (2.872 ± 0.4981)	
		Helioseal F (1.859 ± 0.2288)	-	
		Ultraseal XT (0.9250 ± 0.9547)	-	
		Dyract Seal (0.07714 ± 0.1459)	-	
		**Aged two weeks**	**Aged two weeks**	
		Conseal F (2.915 ± 0.06325)	Control (3.165 ± 0.3695)	
		Helioseal F (3.140 ± 0.1963)	-	
		Ultraseal XT (2.327 ± 0.197)	-	
		Dyract Seal (0.1025 ± 0.00276)	-	
		**Aged one month**	**Aged one month**	
		Conseal F (3.149 ± 0.307)	Control (2.888 ± 0.2604)	
		Helioseal F (3.835 ± 0.1181)	-	
		Ultraseal XT (2.914 ± 0.1369)	-	
		Dyract Seal (2.880 ± 0.2658)	-	
**Bacterial Leakage Testing**	Zmener et al., 2019 [29]	**Leakage frequency after 90 days (*n*)**	**Leakage frequency after 90 days (*n*)**	For the leakage frequency there was no significant difference between EWBMCP and CLPR sealants (*p* > 0.05). However, both showed a significant difference in leakage frequency in comparison to EWB sealant (*p*< 0.05). The EWBMCP sealant showed a higher median survival time in comparison to the other sealants.
		**EWBMCP** 4 out of 10	**EWB** 6 out of 9	
		-	**CLPR** 5 out of 10	
		**Median survival time (absence of bacterial leakage) (days)**	**Median survival time (absence of bacterial leakage) (days)**	
		**EWBMCP** 85.3	**EWB** 72.4	
		-	**CLPR** 80.7	
**Genomic Profiling**	Ibrahim et al., 2020 [21]	-	-	The sealant containing DMAHDM + NACP showed reduction of the relative abundances of the 16S rRNA at the genus level of Streptococcus for both types of inoculum.
**Confocal Laser Scanning Microscopy (CLSM)**	Ibrahim et al., 2020 [21]	-	-	The sealant containing DMAHDM + NACP showed substantial reduction in the formation, distribution and development of the biofilm in both high and low caries-risk pediatric patients in comparison to the control group.
	Ibrahim et al., 2019 [27]	-	-	The sealant containing 5% DMAHDM + 20% NACP showed reduction in visible biofilm biomass in comparison to the experimental control, which showed denser and thicker biofilm.
	Yu et al., 2016 [30]	-	-	The sealant containing 4% MAE-DB showed lower density of cells and greater proportions of dead bacteria in comparison to the controls.
	Tran et al., 2013 [13]	For ***S. mutans***	For ***S. mutans***	The 0.25% containing Se sealant did not show in growth in comparison to the control.
		0.25% selenium	selenium-free sealant	
		Biomass 0	Biomass 315 μm^3^/μm^2^	
		average thickness 0	average thickness 429 μm	
		surface area 0	surface area 47 × 106 μm^2^	
**Scanning Electron Microscopy (SEM)**	Cocco et al., 2020 [10]	-	-	The SnM 5% containing sealant showed reduction in the biofilm total biomass with minimum amount of exopolysaccharides (EPS) in comparison to the control groups.
	Hamilton et al., 2014 [33]	-	-	There was not a significant difference between N6 and CH fiber diameter (*p* = 0.0601).

SD: Standard Deviation, (-): No definitive values were reported.

**Table 5:** Antibacterial effects of dental sealants measuring the metabolic activity, lactic acid production, pH, polysaccharide production and acid stress and oxygen stress tolerance.

Antibacterial Effect				
Assessment	Study	Intervention (Mean ± SD)	Control (Mean ± SD)	Summary of Results
**Metabolic Activity**	Ibrahim et al., 2020 [21]	-	-	The sealants containing 5% DMAHDM + 0% NACP showed significant reductions in metabolic activity in saliva-derived biofilm from both high and low caries-risk pediatric patients in comparison to the control (*p* < 0.05). However, the sealant containing DMAHDM + NACP showed less reduction in comparison to the sealant containing only DMAHDM (*p* < 0.05). There was no significant difference in the same group regarding the type of the saliva inoculum (*p* > 0.05).
	Ibrahim et al., 2019 [27]	-	-	The sealants containing 5% DMAHDM with or without NACP showed significant reductions (82–87%) in metabolic activity in comparison to the other sealants (*p* < 0.05).
	Yu et al., 2016 [30]	-	-	The sealant containing 4% MAE-DB showed significant reduction in metabolic activity in comparison to the controls before and after aging (*p* < 0.05).
**Lactic Acid Production**	Ibrahim et al., 2020 [21]	-	-	The sealant containing DMAHDM + NACP showed reduction of the relative abundances of the 16S rRNA at the genus level of Streptococcus for both types of inoculum.
	Ibrahim et al., 2019 [27]	-	-	The sealants containing 5% DMAHDM with or without NACP showed significant reduction in lactic acid production in comparison to the other sealants (*p* < 0.05).
**pH**	Coco et al., 2020 [10]	***S. mutans***	***S. mutans***	There was no significant difference between the pH of the biofilm cultured on the sealants containing 2.5% and 5% ZnM in comparison to the control group. There was a slight significant difference between 2.5% SnM and the control group The 5% SnM containing sealant kept the pH level close to the neutral.
		2.5% ZnM (4.6 ± 0.0)	Control (4.5 ± 0.0)	
		5% ZnM (4.7 ± 0.1)	-	
		2.5% SnM (5.4 ± 0.1)	-	
		5% SnM (6.7 ± 1.0)	-	
		***S. oralis***	***S. oralis***	
		2.5% ZnM -	Control (5.7 ± 0.9)	
		5% ZnM -	-	
		2.5% SnM -	-	
		5% SnM (6.5 ± 0.4)	-	
		***C. albicans***	***C. albicans***	
		2.5% ZnM -	Control (6.8 ± 0.1)	
		5% ZnM -	-	
		2.5% SnM -	-	
		5% SnM (6.8 ± 0.1)	-	
		***S. mutans*** **and** ***C. albicans***	***S. mutans*** **and *C. albicans***	
		2.5% ZnM -	Control (4.4 ± 0.1)	
		5% ZnM -	-	
		2.5% SnM -	-	
		5% SnM (6.6 ± 0.3)	-	
	Ibrahim et al., 2019 [27]	-	-	There was a significant difference between the pH of the NACP-containing groups in comparison to the other groups at 8-h time point (*p* < 0.05). The NACP-containing groups kept the pH level close to neutral pH at all time points.
**Poly-saccharide Production**	Ibrahim et al., 2019 [27]	-	-	The sealants containing 5% DMAHDM with or without NACP showed significant reduction in polysaccharide production in comparison to the other sealants (*p* < 0.05).
**Acid Stress and Oxygen Stress Tolerance**	Ibrahim et al., 2019 [27]	-	-	The sealants containing 5% DMAHDM showed a lower survival rate at 10 min (38–44%) in comparison to the control and NACP only groups (60–65%) after exposure to pH 2.8. There was no pronounced difference between the groups at the later time points. The sealants containing 5% DMAHDM showed a lower survival rate at 10 and 20 min but not at 30 and 45 min in comparison to the other groups after exposure to 0.2% H_2_O_2_.

SD: Standard Deviation, (-): No definitive values were reported.

## Data Availability

No new data were created or analyzed in this study. Data sharing is not applicable to this article.
